# P-1647. Prevalence of Unnecessary Antibiotic Prescribing for Acute Respiratory Illnesses in U.S. Outpatient Settings, 2022

**DOI:** 10.1093/ofid/ofae631.1813

**Published:** 2025-01-29

**Authors:** Tessa Schwarze, Emily McDonald, Christine Kim, Sarah Kabbani, Adam Hersh, Lauri A Hicks

**Affiliations:** Chenega Enterprises Systems & Solutions, Washington DC, District of Columbia; Centers for Disease Control and Prevention, Cincinatti, Ohio; Centers for Disease Control and Prevention, Cincinatti, Ohio; Centers for Disease Control and Prevention, Cincinatti, Ohio; University of Utah, Salt Lake City, UT; Centers for Disease Control and Prevention, Cincinatti, Ohio

## Abstract

**Background:**

Antibiotics are commonly prescribed for acute respiratory illnesses (ARIs) in outpatient settings, yet up to 50% of antibiotics prescribed for ARIs are unnecessary. We aimed to estimate the prevalence of outpatient antibiotic prescribing for ARIs with no clinical indication for antibiotics (“No Antibiotics Indicated ARIs” or “NAI-ARIs”) and compare it to previously published estimates.

**Figure:**
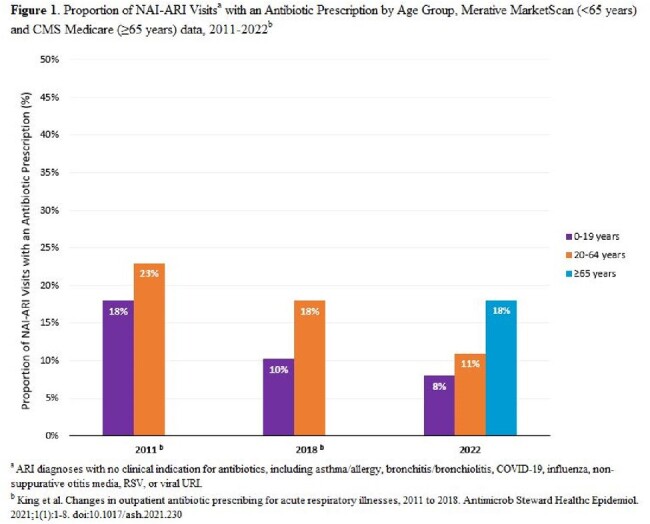

**Methods:**

Patients ages 0–64 years and ≥ 65 years with medical and prescription drug coverage were identified from Merative MarketScan commercial claims and Medicare Part B claims and Part D event files, respectively. We included outpatient visits in 2022 associated with a NAI-ARI diagnosis of asthma/allergy, bronchitis/bronchiolitis, COVID-19, influenza, non-suppurative otitis media, respiratory syncytial virus (RSV) or viral upper respiratory infection (URI). We calculated the visit rate per 1,000 patient-years and proportion of visits resulting in an oral antibiotic for each diagnosis stratified by age group and outpatient setting. The proportion of unnecessary prescribing for individuals ages < 65 years was compared to published proportions from 2011 and 2018; no comparable prior estimates are available for adults ages ≥ 65 years.

**Figure:**
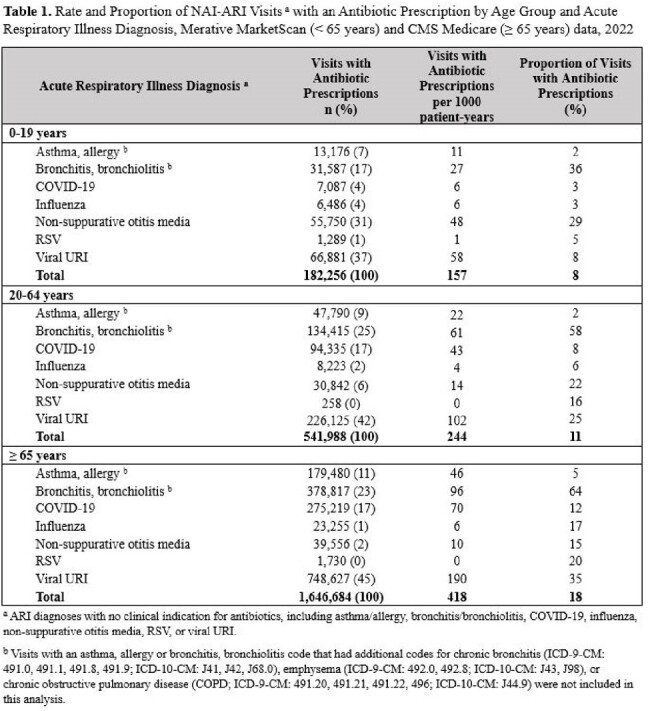

**Results:**

The proportion of NAI-ARI visits resulting in an antibiotic was 18% for adults ages ≥ 65 years, 11% for adults ages 20–64 years, and 8% for children ages 0–19 years. The overall proportion of prescribing for individuals ages < 65 years was lower in 2022 compared to both 2011 and 2018 (**Figure 1**). Across all age groups, bronchitis/bronchiolitis had the highest proportion of visits with an antibiotic, while viral URI had the highest rate of visits with an antibiotic (**Table 1**). Urgent care settings had the highest proportion of visits with an antibiotic across outpatient settings, irrespective of age (**Figure 2** and **Figure 3**).

**Figure:**
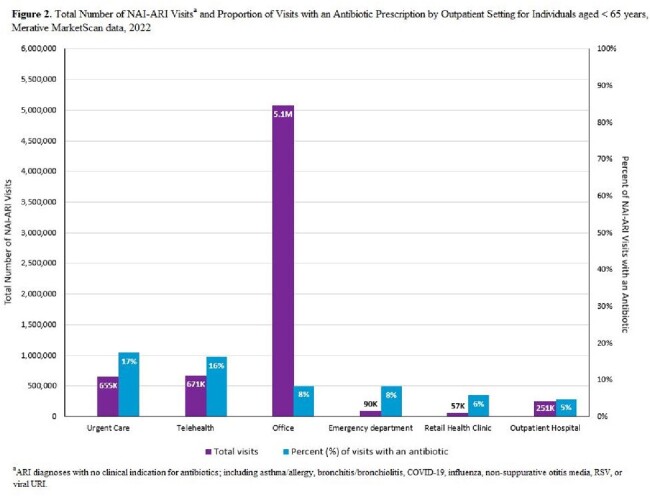

**Conclusion:**

Among patients ages < 65 years, the proportion of unnecessary antibiotic prescribing for NAI-ARIs declined over the past decade, possibly due to antibiotic stewardship activities. Continued efforts to improve outpatient antibiotic use should focus on viral URI, bronchitis/bronchiolitis, and emphasize reducing unnecessary prescribing in older adults and urgent care settings.

**Figure:**
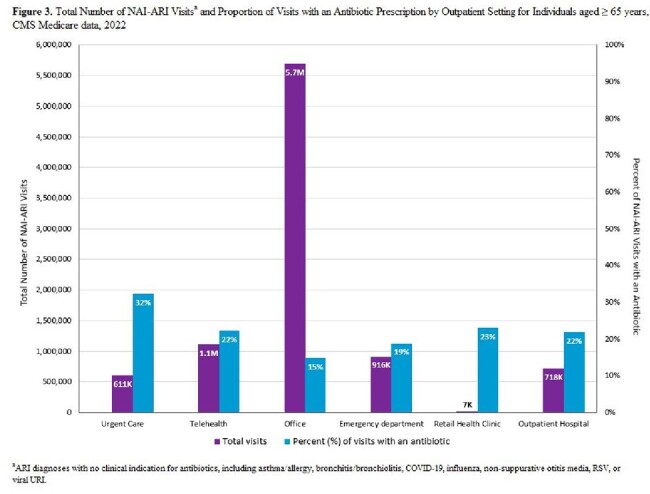

**Disclosures:**

**All Authors**: No reported disclosures

